# ATN plasma biomarkers and digital cognitive performance in adults with type 1 diabetes: data from the GluCog study

**DOI:** 10.1002/alz.093268

**Published:** 2025-01-09

**Authors:** Luciana Mascarenhas Fonseca, Michal S Beeri, Zoë W Hawks, Laneé Jung, Michael Cleveland, Nikki Delgado, Jane Bulger, Elizabeth Grinspoon, Kamille Janess, Martin J. Sliwinski, Ruth S Weinstock, Jasmeer P. Chhatwal, Pia Kivisäkk, Laura Thi Germine, Naomi Sage Chaytor

**Affiliations:** ^1^ Old Age Research Group (PROTER), Department of Psychiatry, University of São Paulo, Brazil, Sao Paulo Brazil; ^2^ Elson S. Floyd College of Medicine, Washington State University, Spokane, WA USA; ^3^ Herbert and Jackeline Krieger Klein Alzheimer's Research Center, Rutgers Biomedical and Health Sciences, Newark, NJ USA; ^4^ Harvard Medical School, Boston, MA USA; ^5^ Institute for Technology in Psychiatry, McLean Hospital, Belmont, MA USA; ^6^ Washington State University, Pullman, WA USA; ^7^ Elson S Floyd College of Medicine, Washington State University, Spokane, WA USA; ^8^ SUNY Upstate Medical University, Syracuse, NY USA; ^9^ Jaeb Center for Health Research, Tampa, FL USA; ^10^ Pennsylvania State University, State College, PA USA; ^11^ Brigham and Women's Hospital, Boston, MA USA; ^12^ Massachusetts General Hospital, Harvard Medical School, Boston, MA USA

## Abstract

**Background:**

Highly specific ATN plasma biomarker assays for neurodegenerative diseases have been developed, but their associations with cognition vary in different populations. Kidney disease, common in diabetes, may decrease the predictive precision of those biomarkers. The aim of this study was to characterize for the first time the relationships between plasma ATN biomarkers and cognitive function in adults with T1D.

**Method:**

Adults with T1D mean age 49 years (range 19‐84), 53% female, mean HbA1c 7.6% who participated in the Glycemic Variability and Fluctuations in Cognitive Status in Adults with T1D study and had plasma β‐amyloid42/40, p‐tau181, neurofilament light chain (NfL) and glial fibrillary acidic protein (GFAP) levels measured using Quanterix, and p‐tau217 using AlzPath assays, were included (N=114). Self‐administered online cognitive tests (TestMyBrain.org) were used: Digit Symbol Matching (processing speed), Gradual Onset Continuous Performance (cognitive control, attention), Multiple Object Tracking (visual working memory); Vocabulary (verbal reasoning), Matrix (non‐verbal reasoning), Simple Reaction Time (psychomotor speed), Letter‐Number Switching (cognitive flexibility), Visual Paired Associates (visual memory), Flicker (working memory), and Paced Serial Addition Test (PSAT, working memory, attention). Bivariate correlation analyses between cognitive function and biomarkers were performed. Significant correlations after false discovery rate were re‐analyzed using linear multiple regression with biomarker assigned as outcome and cognitive function as predictors in separate models, adjusting for age, sex and education.

**Result:**

All biomarkers were correlated with cognitive function in bivariate analysis. Multivariate analyses revealed that higher concentrations of ptau181 and GFAP were associated with longer reaction time for correct responses, PSAT (β=0.002, *p*=0.011 and β=0.057, *p*=0.012, respectively), and higher β‐amyloid42/40 concentration was associated with better vocabulary performance (β=0.028, *p*=0.009). Associations remained significant after including kidney‐related disease or other diabetes features as adjustments.

**Conclusion:**

PTau181, GFAP and β‐amyloid42/40 were associated with cognitive performance in adults with T1D. Working memory speed was related to ptau181 and GFAP, suggesting it may be an early indicator of neurodegeneration in this population. The positive association between β‐amyloid42/40 and Vocabulary, a measure of crystalized cognitive ability, suggests protective mechanisms related to cognitive reserve. Investigation on ATN biomarkers and longitudinal cognitive decline is crucial for disentangling their role in T1D.

